# Fabrication of Graphene Based Durable Intelligent Personal Protective Clothing for Conventional and Non-Conventional Chemical Threats

**DOI:** 10.3390/nano11040940

**Published:** 2021-04-07

**Authors:** Youngho Jin, Dongwon Ka, Seongon Jang, Deokjae Heo, Jin Ah Seo, Hyunsook Jung, Keunhong Jeong, Sangmin Lee

**Affiliations:** 1 4th R&D Institute, Agency for Defense Development, 6th Directorate, Daejeon 34186, Korea; ka0121@add.re.kr (D.K.); ondol0809@gmail.com (S.J.); seoja09@add.re.kr (J.A.S.); junghs@add.re.kr (H.J.); 2 School of Mechanical Engineering, Chung-Ang University, Seoul 06911, Korea; ejrwo472@naver.com; 3 Department of Chemistry, Korea Military Academy, Seoul 01805, Korea; doas1mind@gmail.com

**Keywords:** non-conventional threats, graphene, e-fabric, graphene transfer, triboelectric nanogenerator, chemical sensor, DFT calculation

## Abstract

Conventional or non-conventional chemical threat is gaining huge attention due to its unpredictable and mass destructive effects. Typical military protective suits have drawbacks such as high weight, bulky structure, and unpredictable lifetime. A durable, light, and scalable graphene e-fabric was fabricated from CVD-grown graphene by a simple co-lamination method. The sheet resistance was below 1 kΩ/sq over the wide surface area even after 1000 bending cycles. A graphene triboelectric nanogenerator showed the peak V_OC_ of 68 V and the peak I_CC_ of 14.4 μA and 1 μF capacitor was charged successfully in less than 1 s. A wearable chemical sensor was also fabricated and showed a sensitivity up to 53% for nerve chemical warfare agents (GD). DFT calculations were conducted to unveil the fundamental mechanisms underlying the graphene e-fabric sensor. Additionally, protection against chemical warfare agents was tested, and a design concept of graphene-based intelligent protective clothing has been proposed.

## 1. Introduction

There are growing demands for protecting individuals from conventional and non-conventional threats such as chemical and biological agents. Current CBR (chemical, biological and radiological) personal protective suits for military and emergency responders are classified as activated carbon-based permeable (sorptive) technologies and impermeable barrier technologies [1]. They are selected in different operational conditions such as conventional/non-conventional warfare, terrorist attacks, chemical leak events according to expected types/level of threats, and activity time. Especially, protection from weapons of mass destruction (WMD) is a major concern because the first responders or military personnel rely heavily on PPE (personal protective equipment) [2]. However, the heavy weight, uncomfortable feeling of wearing, reduced mobility, agonizing sensation, and unpredictable lifetime are major drawbacks of current technologies [3]. It is important to develop advanced light weight and multifunctional PPE for military and first responders.

Graphene and its derivatives can be excellent candidates as protective clothing with multi-functionality such as chemical protection, wearable sensing, flame resistance, and energy harvesting. Graphene has been demonstrated in many applications in the past decade, such as photonics [4,5,6], electronics [7,8], energy-related applications [9,10], thermal applications [11], io-applications [12,13,14], and several other applications [15,16]. However, the practical usage of graphene requires the mass production of high-quality graphene and reliable transfer methods for device fabrication [17,18,19]. Industrial applications of high-quality graphene are still a challenge because of the lack of universal, reliable, and economic transfer methods, despite extensive efforts worldwide in the past decades [20,21,22,23,24,25].

In this study, we fabricated a durable and multifunctional graphene e-fabric for PPE against chemical and biological threats. The graphene on the catalytic metal (Cu foil) was directly put in contact with a transparent and flexible hot melt adhesive layer. The opposite side of the adhesive layer was simultaneously attached to target substrates in the commercial roll laminator. Then, the Cu foil was chemically removed to finish the process. The large-area graphene was successfully transferred onto the adhesive layer, which is strongly bonded to PET and fabric substrates. The graphene fabric exhibited excellent electrical conductivity, comparable with that of graphene indirectly transferred on SiO_2_/Si wafers with much better mechanical stability. The mechanical stability of the graphene fabric was tested in the custom-made bending machine over 1000 bending cycles. A triboelectric nanogenerator was fabricated which demonstrated a practical usage of graphene e-textile. A fabric sensor based on graphene for chemical warfare agents was also demonstrated to show practical usage of the graphene fabrics in multifunctional protective clothing. In addition, protection against chemical warfare agents and aerosol was also tested and evaluated.

## 2. Materials and Methods

### 2.1. CVD Graphene Growth

Large-area graphene was grown on commercially available Cu foil (99.9% purity, 35 μm thick) from Graphene Square Inc (Suwon, South Korea). A high-quality monolayer graphene was grown on Cu foil (Graphene Square Inc., Suwon, South Korea) using an inductively coupled plasma chemical vapor deposition (ICP-CVD) system (Graphene Square Inc., Suwon, South Korea). The Cu foil was cut into a 10 cm diameter quartz tube furnace and heated to 1000 °C for 1 h with 10 sccm of H_2_. Then, it was maintained for 30 min with 100 sccm of CH_4_ to grow the monolayer graphene. At the same time, RF (13.56 MHz) plasma power (400 W) was applied to the sample remotely. The initial and working pressures were 1 × 10^−3^ torr and 1.4 torr, respectively. 

### 2.2. Fabrication of Graphene E-Fabric for Multifucitonal Protective Clothing

**Figure 1:**
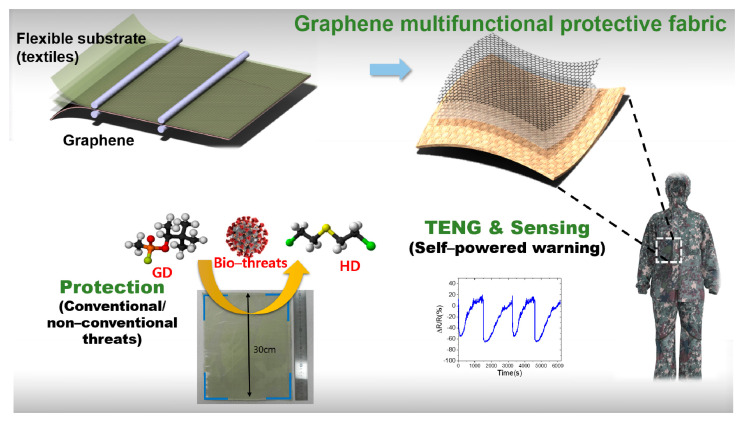
Schematics of graphene multifunctional protective fabrics.

High-quality CVD graphene was directly used for transfer without any surface treatments. The graphene on Cu foil was bonded to hot melt adhesive EVA(Ethylene−vinyl acetate) film (EV504, Chemitec Korea Inc., Seoul, South Korea) while the target substrates, including PET and fabrics, were co-laminated on the other side of the film in the commercial laminator. The temperature of the roll was set to 110–120 °C, approximately 20–40 °C higher than the melting temperature of EVA. A temperature lower than 110 °C was not sufficient for the strong adhesion of the laminates. The melting viscosity of EVA film was 650 mPa∙s at 160 °C, according to the manufacturer. The as-grown graphene on Cu foil showed an almost flat surface; however, it was important to ensure conformal contact between the graphene, EVA, and the substrates. For stable and conformal adhesion between these layers, a PET support film was stacked outside of the target substrate during lamination and then removed. The PET film (Graphene Square Inc., Suwon, South Korea), with a thickness of 100 μm, supported smooth contact between the layers and improved the uniform and strong adhesion. The binding between the layers can be adjusted according to the thickness of the EVA film. It was found that almost any substrates with high-surface roughness formed strong bonds to the EVA film of thickness less than 30 μm. The lamination speed was 30 cm/min. The Cu foil of the laminate was etched in an etching bath (3 wt% ammonium persulfate aqueous solution (Sigma Aldrich Korea, Seoul, South Korea)), then the samples were washed with DI water and dried. Figure 1 shows the fabrication procedure and concept of graphene protective clothing with multi-functionality.

### 2.3. Characterization of Graphene E-Fabric 

The morphology of the transferred graphene was characterized by atomic force microscopy (AFM XE-7, Park systems, Suwon, South Korea) and scanning electron microscopy (SEM, JSM-IT500HR, JEOL, Tokyo, Japan). The roughness of substrates and transferred graphene was investigated using confocal microscopy (OLS 4100, Olympus, Tokyo, Japan). The quality of the CVD-grown graphene transferred onto the SiO_2_/Si wafer was evaluated using Raman spectroscopy (RabRAM HR, Horiba, Kyoto, Japan) with a laser excitation wavelength of 514 nm and XPS (K-alpha, Thermo Scientific, Waltham, MA, USA). The transmittance of the transferred graphene was measured using a UV-Vis spectrometer (UV-2600, Shimadzu, Kyoto, Japan). The electrical properties of the as-transferred graphene were analyzed using a source meter (2401, Keithley, Beaverton, OR, USA) with a 4-point probe station with probe spacing of 1 mm. The mechanical stability of graphene transferred onto fabrics was characterized using a custom-made bending tester. The electrical conductivity of the transferred graphene was measured while performing bending tests over 1000 bending cycles. 

### 2.4. Fabrication and Characterization of Graphene E-Fabric Based TENG and Chemical Sensors

Graphene e-textile based triboelectric nanogenerator was composed of two parts: the upper part and the lower part. First, 8 × 8 cm graphene transferred fabric was attached to a 10 × 10 cm commercial acrylic plate, which acted as the lower part. Next, 7 × 7 cm commercial aluminum tape (thickness of 0.05 mm, DUCKSUNG Co., Seoul, South Korea) was attached to a 10 × 10 cm commercial acrylic plate and 8 × 8 cm commercial PTFE tape (thickness of 0.08 mm, Chukoh Chemical Industries Co., Seoul, South Korea) fully covered on the Al tape. Then, this was used as the upper part. Each lower and upper part was supported by four springs and kept a gap distance of 3 mm. When vertical excitation was applied, the device repeated the contact and separation process. The vertical excitation was provided by a vibration tester (ET-126B-4, Labworks Co., Gwangju, South Korea), a function generator (AFG3021C, Tektronix Co., Beaverton, OR, USA), and an amplifier (pa-151, Labworks Co., Gwangju, South Korea). The vibration amplitude and frequency were set as 6 mm and 10 Hz, respectively. The voltage and current output were measured using an oscilloscope (MDO 3014, Tektronix Co.) and a preamplifier (MODEL SR570, Stanford Research Systems Co., Sunnyvale, CA, USA). The voltage probe 1 and 2 were connected to Al of the upper part and graphene of the lower part, respectively. The voltage output was calculated by subtracting the electric potential of probe 2 from that of probe 1.

Graphene e-fabric based chemical sensors were fabricated. Cu electrodes were coated on graphene e-fabric by photolithography. After stabilizing the responses under ambient air, the graphene e-fabric sensor was exposed to a drop (1 μL) of chemical warfare agents. To test the sensor responses, a constant current (10 μA) was applied to the sensor using a source meter (2401, Keithley, Beaverton, OR, USA), and the voltage drop across the sensors was measured.

### 2.5. Theoretical Study Details

The interaction of adsorbed GD on graphene was studied using DFT calculations. First, the graphene flake structure with hydrogen-terminated, which was large enough to demonstrate the adsorption of GD, was relaxed using B3LYP functional with 6-31 + G(d) basis set. Once optimized, the coordinates of the graphene flake were fixed to represent the whole structure of graphene. Additional optimization, together with frequency calculation of GD on the fixed model structure of graphene, was performed using the Gaussian 16 program. To calculate the adsorption energy, the single-molecule of GD was also optimized by using the same method. After all calculations, minimum structures were confirmed by checking all positive frequencies. Further analysis on molecular orbital composition by the Hirshfeld method was carried out using the Multiwfn program.

## 3. Results

### 3.1. Structural Properties of Graphene E-Fabric 

**Figure 2:**
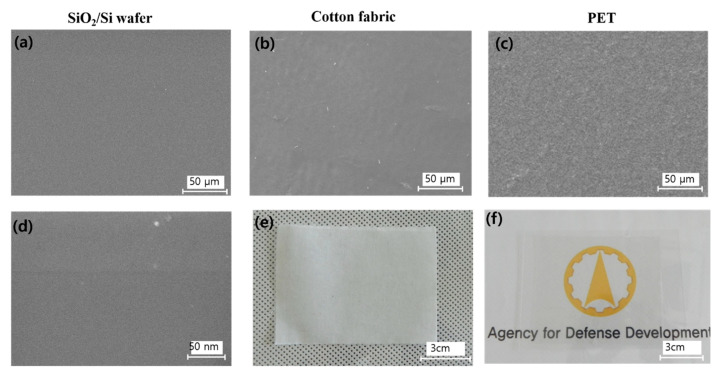
Characterization of graphene on different flexible substrates. (**a**,**d**) SEM images of graphene indirectly transferred on a SiO_2_/Si wafer, (**b**,**e**) SEM and optical images of graphene on cotton fabric, and (**c**,**f**) SEM and optical images of graphene on PET.

**Figure 3:**
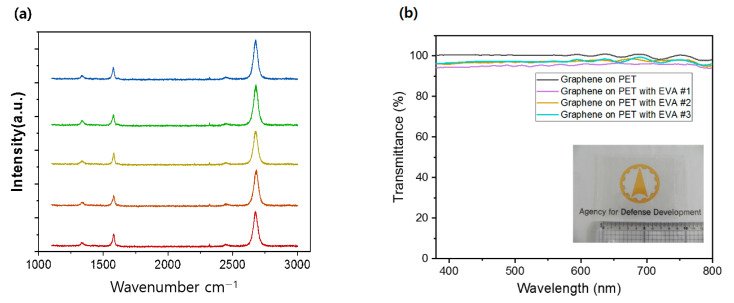
(**a**) Raman spectra of graphene transferred on a SiO_2_/Si wafer by the established PMMA assisted wet transfer method, (**b**) UV-Vis spectra of graphene transferred on EVA(Ethylene−vinyl acetate) with PET.

It was important to verify that the CVD-grown graphene was successfully attached onto fabrics without a high degree of damage or wrinkles. Figure 2 shows SEM and optical images of graphene on different flexible substrates. The macroscopic optical images of the graphene are shown in Figure 2e (cotton fabric) and Figure 2f (PET). There were no macroscopic holes or voids in the wide area of graphene. For comparison, as-grown graphene was indirectly transferred onto a SiO_2_/Si wafer using well-known PMMA supported wet transfer methods [25]. The SEM images of graphene transferred onto the SiO_2_/Si wafer show no significant macroscopic voids, cracks (Figure 2a), nor microscopic damages (Figure 2d). The SEM images of graphene on cotton fabric and PET (Figure 2b,c) show smooth and homogeneous morphology. The graphene exhibited some wavy surface morphology because it bound to the textile substrate with EVA film. There were a few unavoidable wrinkles; however, no macroscopic or microscopic evidence of transfer-induced cracks nor voids were observed. AFM images of graphene on EVA film with PET (Figure S1b) showed an even surface. The confocal microscopy images of graphene on various substrates (Figure S2) also represented conformal contact between the layers. The quality of the synthesized graphene was analyzed using Raman spectroscopy. The Raman spectra of graphene samples on five different locations of the SiO_2_/Si wafer (Figure 3a) showed high-quality monolayer graphene with approximately 2 of 2D/G peak ratio. However, the presence of a small D peak at 1350 cm^−1^ was observed for all samples, representing the grain boundaries of polycrystalline graphene or some inevitable defects.

The quality of graphene after the transfer process was evaluated by electrical and optical properties. In the XPS spectra (Figure S1a), the C1 peak was predominant at 284.4 eV, which represents the sp^2^-hybridized C-C bond of high-quality graphene without residues. UV-Vis spectroscopy was used to verify the transparency of the graphene after the transfer procedure. The transmittance of graphene on EVA with PET is shown in Figure 3b. It was 96.56% for the wavelength range of 380 nm to 800 nm, close to the theoretical value of graphene on quartz [5,26,27]. Although the graphene was mechanically supported by the EVA film, the transparency of pristine graphene was not adversely affected. There was only an approximate 2% decrease in the transmittance from that of graphene on PET without EVA film, which demonstrates that it had no surface damage or contamination during the transfer.

### 3.2. Electrical Properties of Graphene E-Fabric

Electrical conductivity is one of the most important features of graphene-based e-fabrics. Several studies have successfully demonstrated a convenient transfer process with excellent electrical conductivity in the range of 1–10 kΩ/sq [28]. However, the vast majority of previous studies only focused on the electrical conductivity of graphene on PET. Recently, a stamping method was reported for graphene on other polymeric substrates such as PC (polycarbonate), PVC (polyvinyl chloride), and TOPAS (thermoplastic polymer mr-I T85) with comparable electrical conductivity of graphene with green transfer [20]. However, the challenge remains to achieve highly conductive large-area graphene without the aid of surface engineering or even onto real arbitrary substrates. We measured the electrical conductivity of graphene on various flexible substrates, including fabrics. A 4-point probe station with a tip distance of 1 mm was used with a Keithley 2401 source meter. The graphene indirectly transferred on a SiO_2_/Si wafer by the PMMA-supported wet transfer method was also prepared for reference. The graphene samples were successfully attached to PET, cotton fabric, polypropylene (PP) fabric, and even onto polyurethane (PU) foam with EVA film.

**Figure 4:**
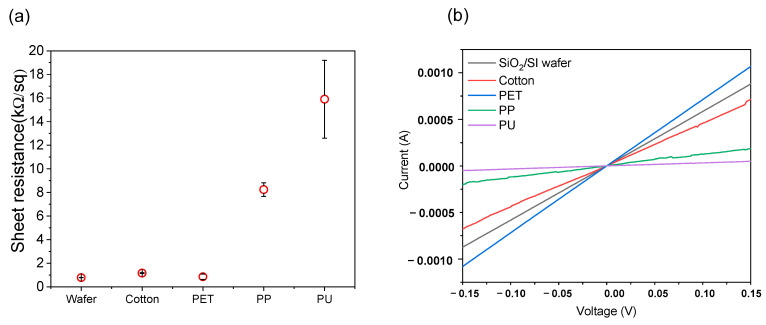
Electrical properties of transferred graphene on EVA with various flexible substrates, (**a**) sheet resistance and (**b**) I-V curves.

Figure 4a shows the sheet resistance of the graphene. The sheet resistance of graphene on the wafer was below 1 kΩ/sq and was homogeneous over a wide area of the surface, equivalent to that of graphene without further doping in the literature [25,27]. The sheet resistances of graphene on cotton fabric and PET also showed values comparable to those of graphene on the wafer. This means that the graphene was not damaged, and its quality was maintained, during the fabrication process, without additional wrinkles, defects, nor surface contamination. The sheet resistance slightly increased when graphene was attached to polypropylene and PU foam, possibly due to the high surface roughness of these materials (Figure S3). Figure 4b shows the I-V curves of the graphene. All the curves showed linear ohmic contacts, and the slope decreased in the order of PET, cotton, PP, and PU substrates, reflecting increased resistance accordingly. 

**Figure 5:**
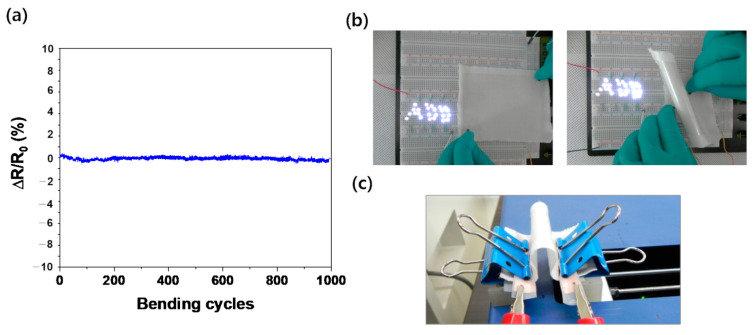
Mechanical stability of transferred graphene on EVA with cotton fabric, (**a**) changes in relative resistance of graphene over 1000 bending tests, (**b**) LED light on through graphene on cotton after 1000 cycles bending test, and (**c**) configuration of bending tests.

The mechanical stability of graphene e-fabric is also important to verify the performance under real operational conditions of protective clothing. The electrical conductivity of graphene was measured while performing bending tests, as shown in Figure 5. The bending induced approximately 6.0% strain (ε_xx_) on the graphene e-fabric. The graphene did not experience noticeable damage or degradation during the bending cycles. The sheet resistance was maintained within ±0.6% over 1000 bending cycles. Moreover, after the bending test, the graphene maintained its resistance as it turned on LEDs. This was because of the strong bond between graphene, EVA film and the cotton fabric. These results confirmed that the graphene e-fabric is mechanically stable and can be used as for e-textiles for wearable sensors, intelligent suits, and chemical protective barriers.

## 4. Discussion

### 4.1. Graphene E-Fabric Based Triboelectric Nanogenerator for Intelligent Protective Clothing

**Figure 6:**
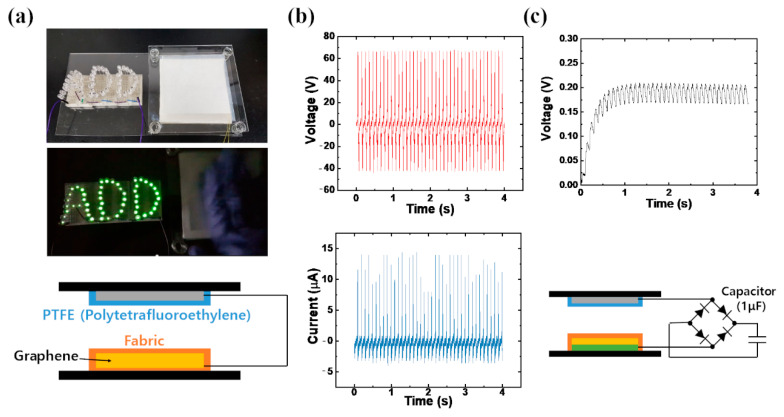
Graphene e-fabric based triboelectric nanogenerator, (**a**) fabrication of TENG, (**b**) open-circuit voltage and closed-circuit current generated at a vertical input frequency of 10 Hz, and (**c**) 1 μF capacitor charged by TENG (circuit illustrated).

The graphene e-fabrics were used in the triboelectric nanogenerator (TENG) to verify its electromechanical properties. The TENG system consists of simple four layers, namely the top electrode, two dielectric layers, and bottom electrode. In this study, polytetrafluoroethylene (PTFE) and commercial cotton fabric were used as dielectric layers. The transferred graphene on the bottom of the cotton fabric was acting as a bottom electrode. As the PTFE layer comes in contact with the cotton fabric layer, triboelectric charges are generated on each contact surface via the triboelectric effect. When the PTFE layer undergoes repeated contact and separation process with cotton fabric, free electrons flow continuously between the top and bottom electrodes via electrostatic induction effect. Figure 6b shows the peak open-circuit voltage and peak closed-circuit current at a vertical input frequency of 10 Hz. As shown in these plots, a maximum peak of 68 V and a peak of 14.4 A were produced, respectively. The electricity generated by the TENG was enough to power 52 LEDs, as shown in Figure 6a. Moreover, the TENG system can charge 1 F capacitor using the rectifying circuit under a vertical vibration input of 10 Hz, as shown in Figure 6c. The capacitor-charging circuit is also illustrated in Figure 6c. The capacitor was charged successfully in less than 1 s. It is believed that the graphene e-fabric can be used in wearable energy-harvesting applications, such as in military uniforms or protective suits. 

### 4.2. Graphene E-Fabric Based Wearable Chemical Sensor for Intelligent Protective Clothing

There are various threats for soldiers in combat environments. Chemical warfare agents (CWAs) can be one of the deadliest threats. Many modern protective suits can offer hours of protection against the liquid or vapor phase of CWA. However, it is also important to warn the soldiers of the presence of CWA and the remaining life of their protective garments or equipment. There are many CWA gas sensors, however, most of them focus on gas threats. Those sensors are used for warning troops, not the individuals. It is highly needed to develop a small, wearable, durable CWA sensor for detecting liquid or aerosol CWA threats. 

**Figure 7:**
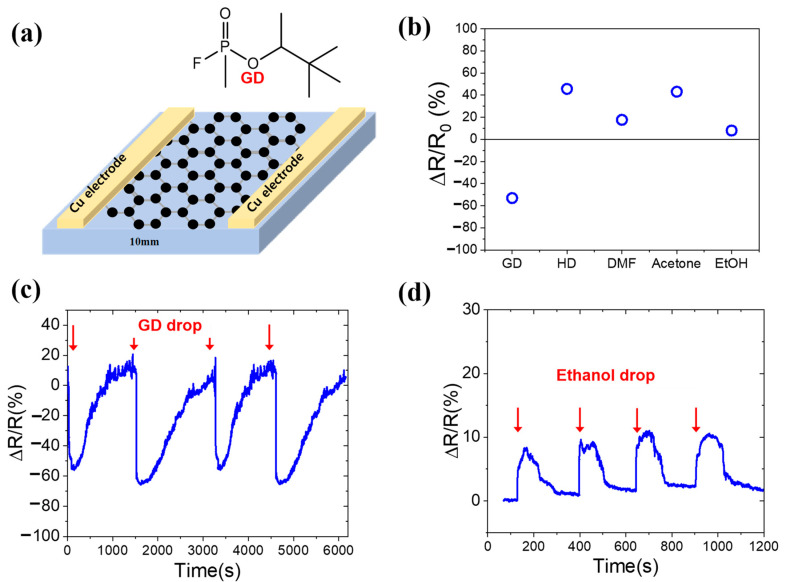
Graphene e-fabric based chemical sensor for detecting chemical warfare agent. (**a**) schematic of the graphene fabric sensor, (**b**) resistance response (ΔR/R) of graphene fabric sensor, (**c**) time-dependent resistance response (ΔR/R) toward liquid GD droplet (2 μL), and (**d**) time-dependent resistance response (ΔR/R) toward ethanol droplet.

The graphene on cotton fabric was used as wearable chemical sensors, as shown in Figure 7a. The electrical resistance was measured with a drop of nerve agent soman (GD). The resistance sensitivity of the graphene fabric sensor was up to −53% for GD while it was 7.9% for ethanol (Figure 7b–d). Furthermore, we confirmed the change of electrical response toward other chemicals such as sulfur mustard (HD), dimethylformamide (DMF), and acetone (Figure 7b). The soman (GD) molecules may work as electron donors (n-type dopant), so they provided electrons to graphene; therefore, the electrical resistivity of graphene is decreased by GD. However, sulfur mustard (HD) may work as an electron acceptor (p-type dopant), so the electrical resistivity of graphene increased by withdrawing electrons from graphene. The graphene fabric chemical sensor fabricated by our transfer method can offer wearable and durable chemical sensors for GD, which can be attachable to personal combat uniforms, protective garments, and many other platforms. It is expected that the selectivity of the sensor can be much improved with proper doping of graphene later. 

### 4.3. DFT Calculations Wearable E-Textile Chemical Sensor Based on Transferred Graphene 

**Figure 8:**
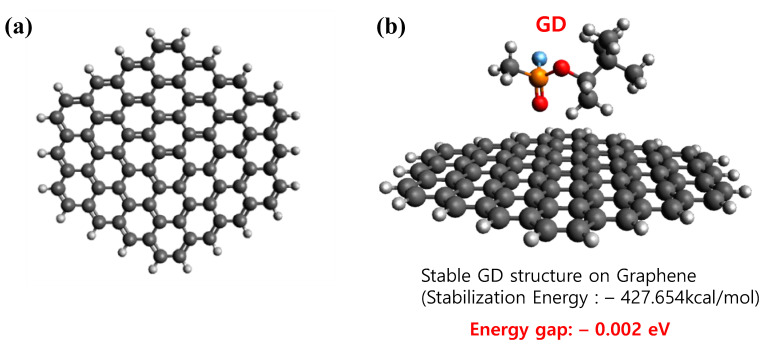
Stable structure before and after the nerve agent GD (soman) is adsorbed in the surface of graphene, (**a**) Simulated hexagonal graphene flakes with hydrogen functionalized edges, (**b**) Adsorption of a GD molecule on graphene.

Density functional theory (DFT) calculations were conducted based on frontier molecular orbital (FMO) theory to understand the electron energy state of graphene in the presence of nerve chemical agent GD (soman). After calculating the ΔE adsorption (E_GD/Graphene_ − (E_GD_ + E_Graphene_)), it was shown that GD was stably and strongly adsorbed on graphene, illustrating its strong effect on the change of electron energy state of graphene by perturbing the orbitals. Its FMO orbital mixing was proved by molecular orbital composition analysis after HOMO and LUMO orbital compositions were computed by the Hirshfeld method [29]. Low but distinct contributions on HOMO (0.011%) and LUMO (0.01%) of GD-adsorbed graphene by oxygen in GD, attributes to the energy gap change of graphene after GD adsorption. The molecular orbital diagram also supported its frontier orbital mixing by GD (Figure S4). According to the energy gap calculation, it was lowered by 0.002 eV after the strong adsorption of GD on graphene, as shown in Figure 8. All this FMO-based analysis after reliable DFT calculation describes the underlying mechanism of the decrease in the resistance of graphene by GD adsorption. 

### 4.4. Protection of Graphene Intelligent Protective Clothing against CWAs, Biological and Radiological Threats

**Figure 9:**
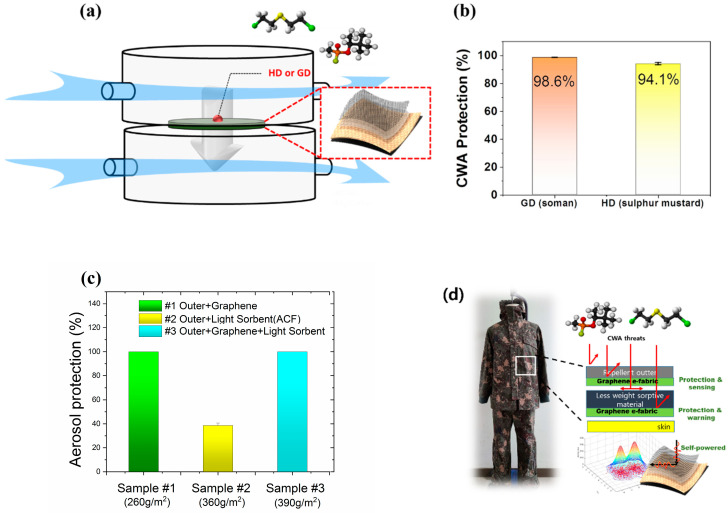
(**a**) Schematic of chemical warfare agent permeation test, (**b**) CWAs protection by graphene fabric, (**c**) 100 nm aerosol protection by protective clothing (#1 Outer layer + graphene, #2 Outer layer + light sorptive layer (activated carbon fiber), #3 Outer layer + graphene + light sorbent (activated carbon fiber), and (**d**) design concept of graphene intelligent multifunctional protective clothing.

To investigate the protective performance of graphene protective clothing, the protection against chemical warfare agents (nerve agent; GD, sulfur mustard; HD) was tested and evaluated. The graphene e-fabric was cut in standard swatch size (4.5 cm in diameter) for liquid challenge/vapor permeation (L/V) test according to military test standard which, is a required part of acquisition testing for the military chemical protective suits. [30] The surface of the graphene e-fabric was contaminated by chemical warfare agents (CWAs) with a medium level of concentration (5 g/m^2^). A stream of conditioned air (32 °C, 80 RH%) was drawn across the top of the swatch, and a clean airflow was drawn across the underside for sampling. This dual flow method causes the agent to diffuse onto the body and skin. The configuration of the CWA permeation test is illustrated in Figure 9a. Interestingly, the graphene e-fabric showed impressive protection against CWAs. The permeated amount of CWAs was only 1.4% for GD and 5.9% for HD (Figure 9b). It should be noted that this was achieved without any liquid-repellent treated outer-shell which is normally used in most current protective suits, and the test fabric was only 40% in weight with respect to the state-of-the-art protective suit currently used by the US Army. 

The protection against viruses, biological agents, and radiological particles was evaluated using 100 nm polystyrene particles (similar in size to SARS-CoV-2). The aerosols were generated with an aerosol generator (model 3073, TSI, US) at 4.5 L/min and neutralized with soft X-ray (SXC-10T, SUNJE electronics, Korea). The number of PS particles was counted by a particle counter (model 3750, TSI, US). The aerosol protection was obtained by Equation (1)

(1) $$\mathrm{Aerosol}\;\mathrm{protection}\;\left(\%\right)=\;\frac{N_{before}-N_{after}}{N_{before}}\times 100$$

Here, *N_before_* is the number of aerosols before filtration by the fabrics, and *N_after_* is the number of aerosols after filtration by the fabrics

The aerosol protection for the fabric, which consists of the outer and the graphene, was almost 100% (Figure 9c). And the weight was only 260 g/m^2^, which was approximately 40% of state-of-the-art protective clothing. In comparison, the aerosol protection for the protective fabric with the outer and a sorptive layer (activated carbon fiber; Zorflex, Chemviron Carbon) was only 38.6%, whereas it can provide decent protection against gaseous CWAs. It is believed that the aerosol protection of CBR protective suits can be much enhanced with the addition of a graphene layer with minor sacrifice in weight.

Based on the properties of graphene e-fabrics, it is possible to suggest multifunctional, intelligent, protective clothing with graphene where it can provide additional protection to the light sorptive materials and self-powered sensing for the presence of chemical threats. It can also warn the wearers of the permeation of threats, or the lifetime of the protective suit with proper positioning of the e-fabric in the layered protective suits, as shown in Figure 9d.

## 5. Conclusions

We have developed a graphene e-fabric based intelligent multifunctional protective clothing for conventional and unconventional threats. The graphene e-fabric showed excellent electrical conductivity with mechanical stability that can be used for chemical protective clothing. A triboelectric nanogenerator and a CWA sensor were fabricated using graphene protective fabric. A DFT calculation was also conducted and explained the underlying mechanisms in the graphene e-fabric CWA sensor. Finally, the protection against chemical warfare agents showed the possibility of graphene-based intelligent, protective clothing with multi-functionality. 

## Data Availability

Data is contained within the article or Supplementary Material.

## Supplementary Materials

The following are available online at https://www.mdpi.com/article/10.3390/nano11040940/s1, Figure S1: (a) XPS spectra of graphene, (b) AFM image of graphene transferred onto EVA film with PET; Figure S2: Confocal microscopy images of as transferred graphene; Figure S3: Electrical properties of as transferred graphene with respect to surface roughness of textiles; Figure S4: FMO diagram (isovalue = 0.001) of GD-adsorbed graphene. Black arrows indicate the oxygen in GD, which contributes each frontier molecular orbital (a) HOMO diagram (b) LUMO diagram.
