# Supplementary material for: Fabrication of Graphene Based Durable Intelligent Personal Protective Clothing for Conventional and Non-Conventional Chemical Threats
Source: Nanomaterials (Basel). 2021 Apr 7;11(4):940. doi: 10.3390/nano11040940 (PMC8067836; doi:10.3390/nano11040940)
Supplement: Supplementary file 1 [file nanomaterials-11-00940-s001.pdf]

Supporting information

Fabrication of Graphene Based Durable Intelligent Personal Protective Clothing for Conventional and Non-Conventional Chemical Threats

Youngho Jin [1,*], Dongwon Ka [1], Seongon Jang [1], Deokjae Heo [2], Jin Ah Seo [1], Hyunsook Jung [1], Keunhong Jeoung [3] and Sangmin Lee [2,*]

1 4th R&D Institute, Agency for Defense Development, 6th Directorate, Daejeon 34186, Korea; ka0121@add.re.kr (D.K.); ondol0809@gmail.com (S.J.); seoja09@add.re.kr (J.A.S.); junghs@add.re.kr (H.J.);

2 School of Mechanical Engineering, Chung-Ang University, Seoul 06911, Korea; ejrwo472@naver.com (D.H.)

3 Department of Chemistry, Korea Military Academy, Seoul 01805, Korea; doas1mind@gmail.com

* Correspondence: cadetnet@add.re.kr (Y.J.); slee98@cau.ac.kr (S.L.); Tel.: +82-42-821-2203 (Y.J.); +82-2-820-5071 (S.L.)

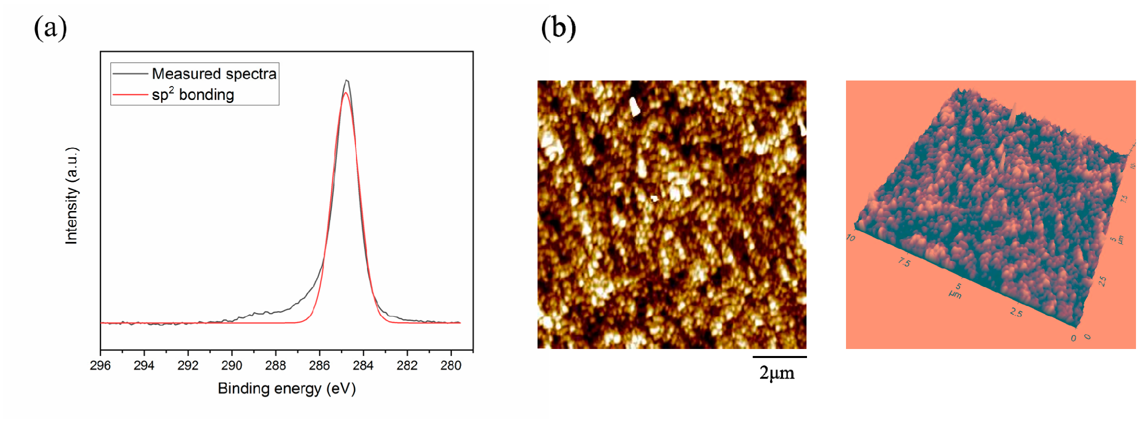

Figure S1 (a)XPS spectra of graphene, (b) AFM image of graphene transferred onto EVA film with PET

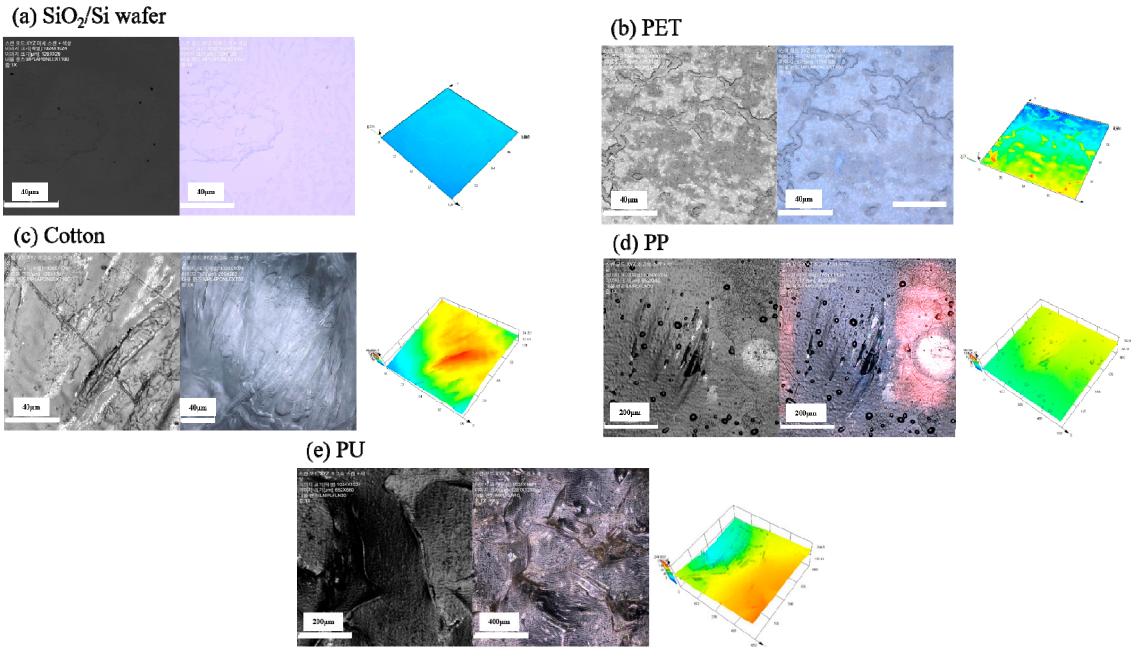

Figure S2. Confocal microscopy images of as transferred graphene.

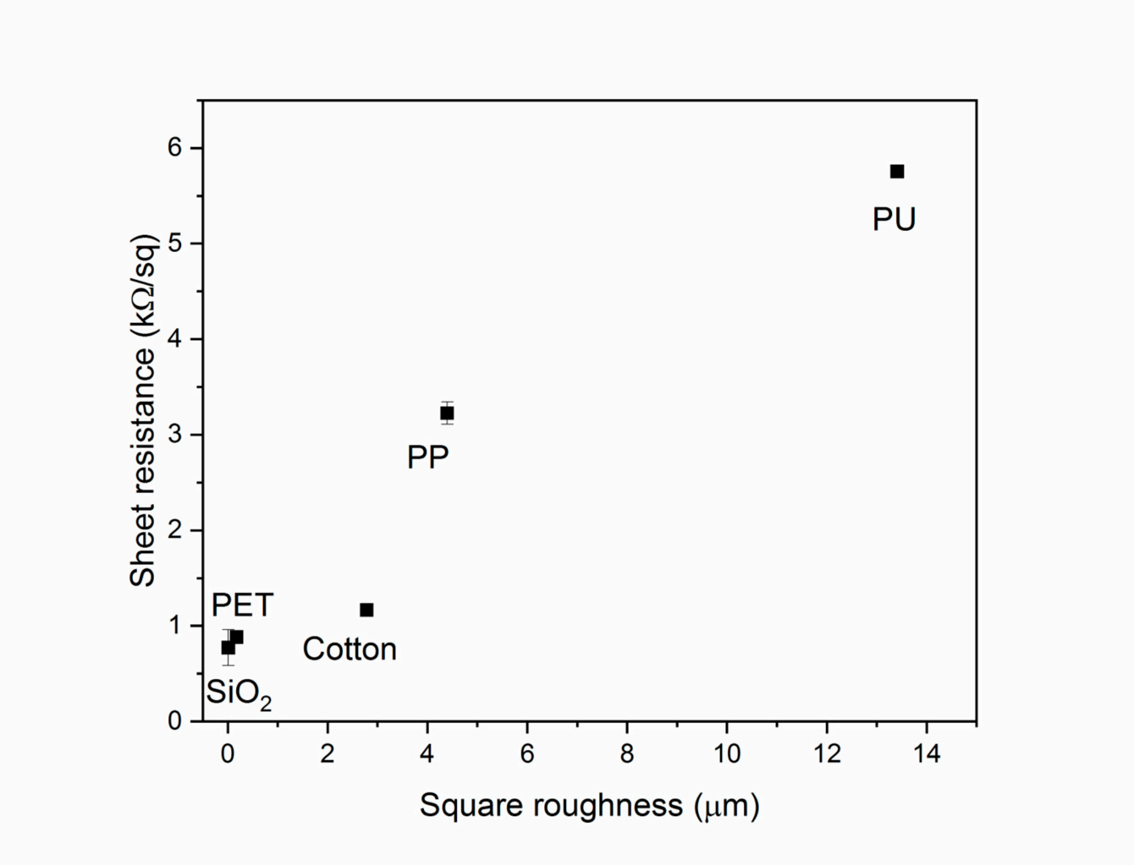

Figure S3. Electrical properties of as transferred graphene with respect to surface roughness of textiles

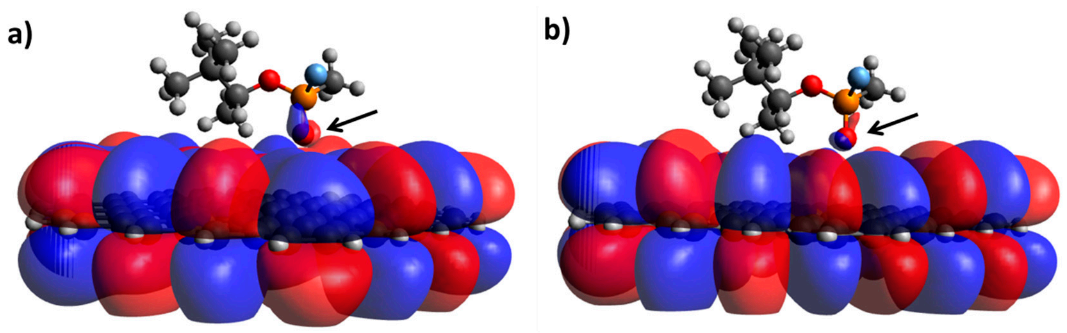

Figure S4. FMO diagram (isovalue = 0.001) of GD-adsorbed graphene. Black arrows indicate the oxygen in GD, which contributes each frontier molecular orbital (a) HOMO diagram (b) LUMO diagram
